# Short bowel syndrome–associated intestinal failure patient experience: A mixed‐method study leveraging an online patient community

**DOI:** 10.1002/ncp.70002

**Published:** 2025-08-07

**Authors:** Brian Po‐Han Chen, Josh Feldman, Megan Gower, Michelle Kirby, Brian Terreri, Maggie McCue, Manpreet S. Mundi

**Affiliations:** ^1^ Inspire Arlington Virginia USA; ^2^ Takeda Pharmaceuticals U.S.A., Inc. Lexington Massachusetts USA; ^3^ Division of Endocrinology, Diabetes, Metabolism, and Nutrition Mayo Clinic Rochester Minnesota USA

**Keywords:** burden of illness, caregivers, healthcare disparities, home parenteral nutrition, mental health, quality of life, short bowel syndrome, social determinants of health

## Abstract

**Background:**

Patients with short bowel syndrome–associated intestinal failure (SBS‐IF) require complex, personalized, and multidisciplinary care; however, there are disparities in access. This study measured the impact of unmet needs and treatment burden among patients and their caregivers.

**Methods:**

This cross‐sectional, mixed‐method study included US adults self‐reporting SBS‐IF and receiving home parenteral nutrition for ≥6 months and their caregivers. One‐hour interviews informed the development of a 30‐min survey administered to participants from an online health community.

**Results:**

Among 68 patients, the mean age was 42 years, 79% were female, and 88% were White. Most of the 16 caregivers were female (69%), and younger than 45 years (69%). Of 32 patients (47%) receiving care from an SBS specialist, only 19 (59%) were referred after diagnosis; in 58% of these, referral occurred >6 months after diagnosis. Depression was reported in 44 patients (65%), with 54% not receiving professional mental health care. Financial concerns were common, with 44 patients (65%) unable to work and 40 patients (59%) reporting annual household income of <$50,000. Of 32 patients reporting difficulty paying medical bills, 22 (32%) could not fill prescriptions and 19 (28%) canceled/delayed healthcare provider visits. Social determinant of health challenges were reported by 44 patients (65%). Caregivers' responsibilities impacted their ability to work (50%) and future outlook (63%).

**Conclusion:**

Patients with SBS‐IF face difficulties accessing specialized healthcare, and are at risk of adverse healthcare outcomes, financial hardships, and poor quality of life. Mental health and work/financial issues were common among both patients and caregivers.

## INTRODUCTION

Short bowel syndrome (SBS) is a challenging and multifaceted disease that is also the most frequent cause of intestinal failure (IF).^1^ Although parenteral nutrition (PN) can be lifesaving in SBS‐associated IF (SBS‐IF), certain inherent life‐threatening complications may occur over the long term.^2^, ^3^ Patients with SBS‐IF receiving home PN (HPN) require close monitoring to ensure they are maintaining protein‐energy, fluid, electrolyte, and micronutrient balance.^4^, ^5^ Additionally, complications arising from having a central catheter must be managed, including infection, thrombosis, or mechanical issues,^5^, ^6^ along with hepatobiliary abnormalities, metabolic bone disease, and renal impairment.^3^, ^6^, ^7^

As such, management of SBS is intricate, requiring a multidisciplinary team (MDT) including primary physicians, registered dietitians, gastroenterologists, nurses, and social workers.^7^, ^8^, ^9^ Despite published guidelines, there is a lack of established treatment algorithms and care pathways.^10^, ^11^ The rarity of SBS conditions may further limit the experience of healthcare providers (HCPs) in disease management. Recent studies have shown a need for more HCP awareness and SBS education.^11^ Addressing these unmet educational needs can support timely diagnosis and standardized management practice across specialties.^11^

Management of SBS‐IF also places significant financial constraints on patients and families, affecting employment, wages, and expenses associated with travel, healthcare visits, and medications.^12^, ^13^, ^14^ A multicenter UK‐based study of family members of patients receiving HPN reported that lack of support by health services was associated with moderate to severe caregiver burden.^15^ Caregivers of adults with SBS‐IF experienced a negative impact on their personal lives and loss of work productivity arising from their caregiving responsibilities.^16^ Previous studies have also noted the impact on a caregiver's quality of life (QoL) and mental health.^14^, ^17^ Despite these detrimental effects, limited data exist regarding the consequences of SBS‐IF and HPN reliance on patients and their caregivers.

The primary study objective was to better understand SBS‐IF patients' and caregivers' experiences regarding diagnosis, symptom management, and treatment. The study additionally sought to identify the impact of unmet needs, including care coordination among MDTs, and to understand experiences with shared decision‐making and QoL of patients and caregivers. Considering the chronic nature of SBS‐IF, this study also explored social determinants of health (SDOH) and the impact of disease on patients' and caregivers' mental health.

## METHODS

A noninterventional, cross‐sectional, mixed‐method study of adult patients with SBS‐IF and their caregivers was conducted among US‐based members of the Inspire online community between March and September 2023. The design followed a sequential format starting with 1‐h qualitative interviews among patients who self‐reported SBS and their caregivers using a semistructured discussion guide and conducted via web‐based teleconferencing. Insights from the qualitative interviews informed development of a 30‐min quantitative survey, which also used a validated QoL instrument.^18^

### Study setting and participants

Participants were recruited from the Inspire digital patient health community platform in collaboration with the Oley Foundation. Eligible patients were aged at least 18 years and lived in the United States. Inclusion criteria necessitated self‐reported SBS‐IF requiring HPN. Participants were excluded if they started HPN after September 2022 (6 months before data collection commenced) or were receiving full‐time medical facility care.

### Survey items

The patient survey addressed several categories, including diagnosis of SBS, clinical factors leading to nutrient malabsorption requiring HPN, treatment experience, impact of PN, mental health, and various aspects of SDOH, such as housing, employment, and transportation. The caregiver survey mirrored the patient survey from the caregiver perspective while supplementing information about the caregivers and their relationships with patients. QoL for patients was assessed using the validated Short Bowel Syndrome‐Quality of Life tool (SBS‐QoL), which features 17 items grouped into two subscales.^19^ Subscale 1 scores range from 0 to 110, and subscale 2 yields a score ranging from 0 to 60. Total scores range from 0 to 170, with higher scores indicating worse QoL.

### Ethical compliance

This study received an exemption from Western Copernicus Group Institutional Review Board review under 45 CFR § 46.104(d)(2). Informed consent was obtained digitally from all participants, ensuring confidentiality and minimal risk.

### Data analysis

Thematic transcript content analysis was conducted following the qualitative interviews. Survey data were summarized with descriptive statistics (mean ± SD or percentage) using R version 4.2.1, developed by R Core Team.

## RESULTS

### Phase 1: Qualitative interviews

Six patients with SBS and four caregivers were interviewed; two caregivers were patients' spouses and two were patients' mothers. Most interviewees were female (60%) and had some college education (80%). Half were aged at least 45 years (50%) and most resided in the Southern United States (80%).

Three key themes were identified. First, patients with SBS can experience long, painful, and emotional journeys, with severe cases leading to extended hospital stays, distress from diagnosis, and HPN reliance. Adapting to PN and resultant lifestyle changes is challenging.

Second, SBS profoundly impacts patients' QoL. Lifelong PN dependency, dietary changes, and employment disruptions reduce social and psychological well‐being. Despite these challenges, few patients receive mental health care. The visible nature of SBS, with many patients tethered to an intravenous (IV) delivery system for long periods, adds to extreme mental distress.

Financial burden was the third identified theme. Absence of a specific *International Classification of Diseases 10th Revision* (*ICD‐10*) code for SBS complicated medication approvals from insurers. The patient experiences explored in this study occurred before the addition of an SBS‐specific *ICD‐10* code (K90.82) in October 2024. Before this addition, patients would often rely on assistance programs for out‐of‐pocket costs. Uninsured individuals face significant financial strain.

The quantitative survey was designed to measure the impact of SBS on patients and caregivers based on these themes. It included items assessing the diagnosis journey, impact of symptoms, and perceived effectiveness and satisfaction of PN and other treatments. The survey assessed QoL, impact of PN and nutrient absorption medications, mental health, financial burden, and experiences with SDOH.

### Phase 2: Quantitative survey

Of 68 patients completing the survey, the majority were female (79%), younger than 45 years (63%), and had a body mass index (BMI) of 18.5–24.9 (calculated as weight in kilograms divided by height in meters squared) (62%). The Midwest region was the most represented (31%) (Table 1). Overall, 16 caregivers completed the survey; some were referred by patients who completed the survey. All were close family members and 25% were parents of patients with SBS‐IF. The majority were female (69%) and younger than 45 years (69%).

**Table 1:** Characteristics of patients and caregivers.

Variables	Patients (*n* = 68)	Caregivers (*n* = 16^a^)
Age, mean (SD), years	42.0 (14.9)	39.1 (14.2)
Age, *n* (%), years		
18–24	8 (12)	2 (13)
25–34	12 (18)	4 (25)
35–44	23 (34)	5 (31)
45–54	11 (16)	3 (19)
55–64	5 (7)	1 (6)
≥65	9 (13)	1 (6)
Gender, *n* (%)		
Male	12 (18)	5 (31)
Female	54 (79)	11 (69)
Nonbinary	2 (3)	0 (0)
Ethnicity, *n* (%)		
Hispanic/Latino/Spanish origin	2 (3)	3 (19)
Not of Hispanic/Latino/Spanish origin	66 (97)	13 (81)
Race, *n* (%)^b^		
American Indian or Alaskan Native	0 (0)	0 (0)
Asian	5 (7)	1 (6)
Black or African American	2 (3)	2 (13)
Native Hawaiian or Pacific Islander	1 (1)	0 (0)
White	60 (88)	13 (81)
Other	2 (3)	0 (0)
Highest level of education, *n* (%)		
Some high school, but did not graduate	0 (0)	0 (0)
High school graduate or GED	3 (4)	3 (19)
Some college but less than a bachelor's/undergraduate degree	27 (40)	5 (31)
College bachelor's degree/undergraduate degree	21 (31)	6 (38)
Postgraduate degree (master's, doctorate, etc)	12 (18)	2 (13)
Trade school, professional program	5 (7)	0 (0)
Prefer not to answer	0 (0)	0 (0)
Region, *n* (%)		
Northeast	17 (25)	3 (19)
South	16 (24)	6 (38)
Midwest	21 (31)	4 (25)
West	14 (21)	3 (19)
BMI, *n* (%)		
<18.5	15 (22)	N/A
18.5–24.9	42 (62)	N/A
25–29.9	7 (10)	N/A
30–39.9	3 (4)	N/A
≥40	1 (1)	N/A

*Note*: BMI was calculated as weight in kilograms divided by height in meters squared.

Abbreviations: BMI, body mass index; GED, General Educational Development; N/A, not applicable.

^a^ One caregiver did not provide demographic information beyond gender and location.

^b^ Patients could identify as more than one race.

#### Overall disease burden

Patients reported several challenges in housing, employment, transportation, and caregiver support. Over a quarter of patients expressed housing concerns (29%). Under a quarter of patients had a house that was owned by them or someone in their household with no mortgage (24%). Nearly two‐thirds of patients were medically unable to work (65%) and two were also full‐time caregivers themselves (3%). Most patients received care in an academic center or university hospital (65%). Approximately one‐third of patients relied on family or friends for transportation (32%). Over one‐third of patients spent >1 h traveling for SBS‐related HCP appointments (37%). Most patients reported having caregiver support (93%), with 36 (53%) living with a caregiver. Almost 60% of patients had received care support for >5 years, and the majority of patients received care support for <24 h per week (86%).

Among caregivers, 12% expressed concerns about housing stability. Most caregivers lived in the same household as the patient they cared for (94%), half had been providing care for ≥5 years (50%), and most provided care for <24 h a week (88%). Most caregivers reported that the patient received treatment in a university hospital or academic medical center (63%), and 75% stated that they or another family member were responsible for providing transportation for the patient.

#### SBS causes and diagnosis

Almost half of patients (44%) reported having a formal SBS diagnosis, with 10% of patients unsure. However, among the 38 patients who did not report a formal SBS diagnosis, 32 (84%) reported functional gastrointestinal (GI) disorders, GI surgery or damage, developmental abnormalities, midgut volvulus, or other disorders leading to chronic malabsorption, indicating a high likelihood of lack of formal SBS diagnosis or lack of awareness of SBS diagnosis. Overall, 62 of 68 patients (91%) reported these factors as responsible for their HPN requirement. Approximately half of all patients reported their condition was caused by a functional GI disorder (54%); another 37% reported their condition was induced by surgery to repair damage to the small intestine from underlying disease. Of patients with a formal SBS diagnosis, the majority (53%) had received a diagnosis <10 years ago. Of all patients, 53 (78%) were diagnosed by a gastroenterologist. Most patients were then referred to other HCPs. Of the 53 patients referred to an HCP of a different specialty, 27 (50%) saw a nutritionist and 25 (47%) saw an additional gastroenterologist. Nearly half of all patients (47%) were receiving care from an SBS specialist, with 19 of them (59%) referred after diagnosis, 9 (28%) at the point of SBS diagnosis, and 4 (13%) seeking specialist care on their own.

Half of the caregivers reported functional GI disorders as the primary factor leading to SBS symptoms and diagnosis. Caregivers reported that the most common additional SBS‐related conditions experienced by the patient included central line infections (63%) and trouble maintaining weight or failure to thrive (56%).

#### Perceived effectiveness and experiences with treatments

The most common PN frequency among patients was daily (79%), with 61% receiving PN for >12 h per day. Nearly all patients (93%) received PN at home, and 41% reported deviation from the prescribed HPN infusion plan. As a treatment for SBS, HPN is often supported by other treatments such as IV fluids (IVF) (93%) and enteral nutrition (EN) (75%). Less than 25% of patients were prescribed nutrient absorption medications. Patients who confirmed receiving specific treatments were asked to rate the perceived effectiveness of their current treatments on a scale of 1 (not at all effective) to 7 (extremely effective). HPN, which was received by all participants (*N* = 68), was rated the highest, with a mean ± SD score of 5.5 ± 1.5. EN and nutrient absorption medication were rated as less effective, with mean scores of 3.2 ± 1.9 and 3.0 ± 1.7 (*N* = 10), respectively. Satisfaction with patients' current treatments was also rated on a scale of 1 (not at all satisfied) to 7 (extremely satisfied). Patients were satisfied with IVF and HPN, with mean satisfaction scores of 5.1 ± 1.7 and 5.8 ± 1.4, respectively.

Caregivers were asked to evaluate the patient's SBS treatments and reported similar findings, identifying IVF and HPN as the most effective treatments, with mean scores of 5.8 ± 1.5 and 5.5 ± 1.4, respectively. EN and nutrient absorption medication were perceived as least effective, with mean scores of 4.0 ± 1.3 and 3.5 ± 3.0, respectively. Caregivers reported moderate satisfaction levels across currently used treatments, with highest satisfaction scores for IVF and HPN (mean scores of 5.5 ± 1.9 and 5.1 ± 1.6, respectively), and the lowest satisfaction for nutrient absorption medications (mean score 3.5 ± 2.6). Furthermore, caregivers generally had more positive perceptions than patients about the impact of nutrient absorption medications on domains such as ability to work, travel, and stay active.

#### Impact of treatments on QoL

**Figure 1:**
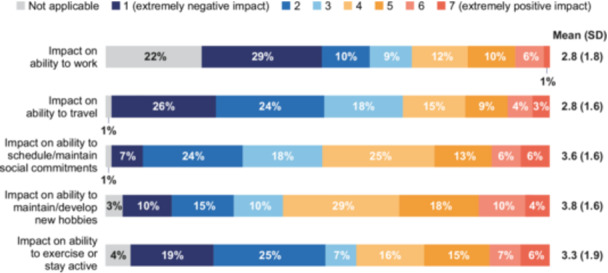
Patient‐reported treatment impact on quality of life.

Patients were asked about the impact of HPN on their daily lives, focusing on their ability to work, travel, maintain social commitments, maintain/develop new hobbies, and exercise. Responses were rated on a scale from 1 (extremely negative impact) to 7 (extremely positive impact). The response was generally negative across domains, particularly affecting patients' ability to work and travel, with mean scores of 2.8 ± 1.8 and 2.8 ± 1.6, respectively (Figure 1). HPN had a slightly less negative impact on maintaining social commitments and developing new hobbies, with mean scores of 3.6 ± 1.6 and 3.8 ± 1.6, respectively. Among patients taking nutrient absorption medication (*n* = 10), only 20%–30% reported positive to extremely positive impacts across the domains.

The mean SBS‐QoL summary score was 118.5 ± 31.6 on a scale of 0 to 170. Most patients (82%) had a score of >100, indicating relatively poor QoL. Stratified analysis of SBS‐QoL score by patient characteristics highlighted important trends (Table 2). Patients with a healthy or overweight BMI (18.5–29.9) had better QoL (mean score 111.0 ± 32.4) than those in underweight (<18.5, mean score 136.1 ± 20.7) or obese (≥30, mean score 144.4 ± 10.0) categories. Moreover, patients with higher annual household income (>$100,000) had better scores (mean score 99.7 ± 26.6) than those with lower annual household income (<$50,000; mean score 127.1 ± 23.1).

**Table 2:** SBS‐QoL score by patient characteristics.

Variable	Patients (*N* = 68)
SBS‐QoL score (continuous), mean (SD)	118.5 (31.6)
SBS‐QoL score (categorical), *n* (%)	
0–100	12 (18)
101–170	56 (82)
**Patient characteristics**	
Gender, mean (SD)	
Male	119.6 (37.4)
Female	118.2 (30.9)
SBS diagnosis history, mean (SD)	
SBS diagnosis	111.8 (39.2)
No SBS diagnosis	123.7 (23.2)
BMI, mean (SD)	
<18.5	136.1 (20.7)
18.5–29.9	111.0 (32.4)
≥30	144.4 (10.0)
Annual household income level, mean (SD)	
<$25,000	127.4 (24.3)
$25,000– < $50,000	126.7 (21.7)
$50,000– < $75,000	121.7 (21.7)
$75,000– < $100,000	104.3 (54.5)
≥$100,000	99.7 (26.6)
Caregiver support, mean (SD)	
Has caregiver	115.7 (36.1)
No caregiver	122.6 (23.1)
Depression support history, mean (SD)	
Has history	123.9 (25.3)
No history	110.0 (41.8)
Current teduglutide use, mean (SD)	
Yes	132.7 (23.0)
No	115.7 (32.4)

Abbreviations: BMI, body mass index; QoL, quality of life; SBS, short bowel syndrome; SBS‐QoL, Short Bowel Syndrome‐Quality of Life Questionnaire.

Most caregivers supported patients with household chores (95%) and attended HCP appointments (95%) with them. Impacts of SBS on caregivers were assessed in multiple domains, including ability to be sexually active with partners/spouse, desire to have children, ability to maintain friendships, and career trajectory/educational plans. The most impacted domain was the ability to be sexually active, with a mean score of 4.0 ± 2.4. Half of caregivers noted a negative impact on their ability to work (50%) and nearly two‐thirds reported their future outlook is at least moderately negatively impacted (63%).

#### SDOH and financial burden

**Figure 2:**
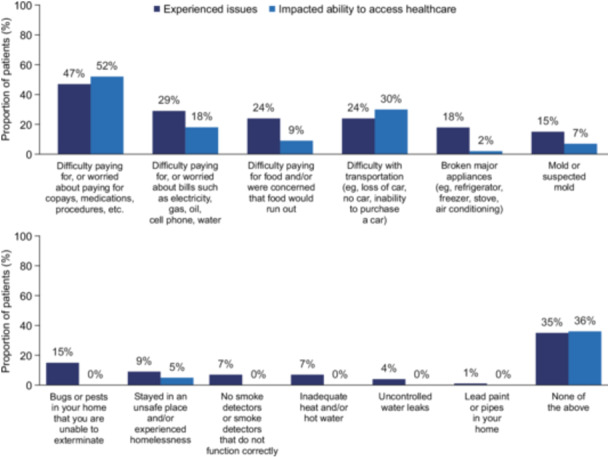
Patient‐reported experiences with social determinants of health. Figures 1 and 2 are reprinted from Gastroenterology, Vol. 166, Issue 5, Feldman J., Raphael B.P., Matthews C., Gower M., Kirby M., Richardson J., Chen B.P., Mundi M., “Tu1961 Short Bowel Syndrome Patient Experience: A Mixed‐Method Study Leveraging Inspire, an Online Community Platform,” Pages S‐1480, Copyright (2024), with permission from Elsevier.

Most respondents (65%) reported challenges related to at least one SDOH. The leading issues experienced were difficulty affording copays, medications, and procedures (47%), followed by difficulty paying for utility bills (29%) and difficulty paying for food (24%) and transportation (24%) (Figure 2). The least common issues were homes having lead paint and water leaks, which affected 1% and 4% of respondents, respectively. Among the 32 patients who had difficulty paying medical bills, 22 (69%) could not fill their prescription on time or at all, 19 (59%) had to cancel/delay HCP visits, and 12 (38%) had to cancel/delay in‐office treatments.

#### Mental health impact

Depression rates were high among patients, with two‐thirds reporting diagnosed or suspected depression (65%). Despite this, only 46% met with mental health professionals. Over one‐third of caregivers were diagnosed with or suspected that they had depression (37%), with the majority not receiving care from a mental health professional (70%).

#### Patient education and shared decision‐making

Only 37% of patients recalled receiving ≥30 min of education about their condition during their first visit. Satisfaction with HCP explanation of SBS and treatment options was low (39%). Most patients reported that the decision to prescribe HPN was shared with the HCP (47%); however, 29% of caregivers reported that the HCP made the decision without patients' input (vs 3% reported by patients). Caregivers preferred high involvement in treatment decisions (57%) and most reported some level of actual involvement in prescribed treatment (79%).

## DISCUSSION

This article presents the findings of a mixed‐method study among US patients self‐reporting SBS‐IF and their caregivers. Most patients reported SBS specialist involvement in their care, and most were referred after diagnosis. For many patients, the average travel time to an HCP for treatment was >1 h. Mental health concerns were prevalent, with most patients reporting depression but lacking mental health support. Financial concerns were common, affecting ability to work and housing security, with annual household incomes mostly below $50,000. Skipped prescriptions and missed HCP visits were frequent. Overall QoL scores were generally low, with better scores linked to healthy/overweight BMI and higher annual household incomes.

This study provides further evidence quantifying patient experiences with SDOH in which most patients had reported having at least one SDOH‐related issue. Although patients and caregivers found HPN more effective than other nutrition modalities, they reported difficulty obtaining an SBS diagnosis, a lack of adequate education about SBS, and difficulty visiting SBS specialists, often having to travel extensively for care. This translated to a significant financial burden on patients and caregivers, contributing to poorer QoL and mental health.

Less than half of the patients reported having formal SBS diagnoses, potentially attributable to the previous unavailability of *ICD‐10* codes and lack of treatment by a formal MDT.^14^ Estimating SBS incidence and prevalence is challenging owing to the rarity and lack of consistency in the application of disease criteria, healthcare database variations, and state‐level healthcare differences.^14^ Previous estimates for SBS prevalence have used HPN prevalence as a proxy. A 2022 study estimated that 24,048 individuals were receiving HPN in the United States between 2012 and 2020.^20^ The reported HPN prevalence estimate was 75 per million in the United States, higher than in other countries.^14^, ^20^ The majority of these were women older than 45 years who were receiving treatment from HCPs inexperienced in treating SBS. Additionally, patients with SBS had to travel long distances for care, especially when traveling from rural areas.^20^

Family and social support systems play an important role in the overall well‐being of patients with SBS. Most families have reported decreased insurance coverage, further impacting their QoL and mental health.^14^, ^17^, ^21^ A recent survey revealed that PN access and reimbursement issues, such as low insurance coverage, increasing out‐of‐pocket costs for medications and supplies, and high insurance premiums, are associated with PN errors and adverse events.^19^ Research also suggests that patients receiving HPN are more exposed to financial situations that may result in the loss of a home, divorce, or medical bankruptcy.^12^, ^17^ Patients living in lower‐income neighborhoods have an even higher prevalence of SBS‐related hospitalization, infections, and longer length of hospital stay.^22^

Most patients reported poor QoL in this study, and treatment was reported to severely affect patients' ability to work and travel. A recent systematic review concluded that the QoL of patients with SBS was lower than that of the general population regarding physical functioning and psychological domains.^23^ A 2023 review determined that the volume and number of PN infusions per week has an inverse relationship with QoL in addition to the known factors negatively impacting QoL, such as diarrhea, pain, nocturia, fatigue, depression, and narcotic dependence.^14^

Living with SBS and HPN dependency impacts patients and their caregivers. Most patients and a high percentage of caregivers in this study reported they were likely to have depression; however, most of them were not receiving mental health care. Financial burden from inability to work also contributes to poor QoL among patients with PN dependency and to their caregivers' mental health status, income adequacy, depression, and fatigue.^14^, ^24^, ^25^

The strengths of this study include use of a mixed‐method approach, in which the survey was developed based on qualitative patient inputs and with an expert gastroenterologist, and the ability to recruit patients virtually across the United States via Inspire's direct patient access and existing partnership with the Oley Foundation to expedite study recruitment. Limitations include potential recall bias with self‐reported information, as with any survey‐based study, as well as the potential for self‐selection bias. Limited sample sizes, particularly among caregivers, may affect generalizability. In addition, given the prior absence of an official SBS diagnosis code (up to *ICD‐10*), patients with SBS may be unaware of the condition and instead identify with chronic nutrient malabsorption and HPN dependency.

## CONCLUSION

The study expands existing knowledge of SBS, demonstrating the physical limitations of SBS and its impact on QoL, SDOH, and mental health for patients and caregivers, enhancing the understanding of the real‐world implications of living with this condition. Longitudinal research is warranted, and online patient health community platforms such as Inspire are uniquely positioned to understand and measure the impact of treatment options over time in this underserved population.

## AUTHOR CONTRIBUTIONS

Brian Po‐Han Chen, Josh Feldman, Megan Gower, Michelle Kirby, Maggie McCue, Brian Terreri, and Manpreet S. Mundi participated in the conceptualization and design of the study. Josh Feldman analyzed the data and provided the methods. Brian Po‐Han Chen, Josh Feldman, Megan Gower, Michelle Kirby, and Manpreet S. Mundi reviewed and verified the data. All authors interpreted the data. All authors critically revised the manuscript, agree to be fully accountable for ensuring the integrity and accuracy of the work, and read and approved the final manuscript.

## CONFLICT OF INTEREST STATEMENT

Brian Po‐Han Chen and Josh Feldman are Inspire employees. Megan Gower, Michelle Kirby, Brian Terreri, and Maggie McCue are employed by Takeda Pharmaceuticals U.S.A, Inc. and receive stock and/or stock options. Manpreet S. Mundi has received research grants from Fresenius Kabi, Nestlé, and Real Food Blends. He is also a consultant for Baxter and part of an emerging experts SBS group with Zealand Pharma, outside of the submitted work.

## Supporting information

inspire dg‐takeda sbs patient journey‐jan 20 2023 final clean NCP v2.

## Data Availability

The datasets, including the redacted study protocol, redacted statistical analysis plan, and individual participants' data supporting the results reported in this article will be made available within 3 months from initial request to researchers who provide a methodologically sound proposal. The data will be provided after deidentification, in compliance with applicable privacy laws, data protection, and requirements for consent and anonymization.

## References

[1] Pironi L . Definitions of intestinal failure and the short bowel syndrome. Best Pract Res Clin Gastroenterol. 2016;30(2):173‐185. 27086884 10.1016/j.bpg.2016.02.011

[2] Fuglsang KA , Brandt CF , Eliasson J , Jeppesen PB . Su2010–Mortality and outcomes in patients with non‐malignant short bowel syndrome receiving home parenteral support. Gastroenterology. 2019;156(6):S–689.

[3] Jeppesen PB , Gilroy R , Pertkiewicz M , Allard JP , Messing B , O'Keefe SJ . Randomised placebo‐controlled trial of teduglutide in reducing parenteral nutrition and/or intravenous fluid requirements in patients with short bowel syndrome. Gut. 2011;60(7):902‐914. 21317170 10.1136/gut.2010.218271 PMC3112364

[4] Kelly DG , Tappenden KA , Winkler MF . Short bowel syndrome: highlights of patient management, quality of life, and survival. JPEN J Parenter Enteral Nutr. 2014;38(4):427‐437. 24247092 10.1177/0148607113512678

[5] Sowerbutts AM , Panter C , Dickie G , et al. Short bowel syndrome and the impact on patients and their families: a qualitative study. J Hum Nutr Diet. 2020;33(6):767‐774. 32779284 10.1111/jhn.12803

[6] Winkler MF , Hagan E , Wetle T , Smith C , Maillet JO , Touger‐Decker R . An exploration of quality of life and the experience of living with home parenteral nutrition. JPEN J Parenter Enteral Nutr. 2010;34(4):395‐407. 20631385 10.1177/0148607110362582

[7] Pironi L , Boeykens K , Bozzetti F , et al. ESPEN practical guideline: home parenteral nutrition. Clin Nutr. 2023;42(3):411‐430. 36796121 10.1016/j.clnu.2022.12.003

[8] Matarese LE , Jeppesen PB , O'Keefe SJD . Short bowel syndrome in adults: the need for an interdisciplinary approach and coordinated care. JPEN J Parenter Enteral Nutr. 2014;38(suppl 1):60S‐64S. 24418899 10.1177/0148607113518946

[9] Hofstetter S , Stern L , Willet J . Key issues in addressing the clinical and humanistic burden of short bowel syndrome in the US. Curr Med Res Opin. 2013;29(5):495‐504. 23480444 10.1185/03007995.2013.784700

[10] Staun M , Hebuterne X , Shaffer J , et al. Management of intestinal failure in Europe. A questionnaire‐based study on the incidence and management. Dyn Med. 2007;6(1):7. 17610741 10.1186/1476-5918-6-7 PMC1945021

[11] Belcher E , Mercer D , Raphael BP , Salinas GD , Stacy S , Tappenden KA . Management of short‐bowel syndrome: a survey of unmet educational needs among healthcare providers. JPEN J Parenter Enteral Nutr. 2022;46(8):1839‐1846. 35511707 10.1002/jpen.2388 PMC9790246

[12] Winkler MF , Smith CE . Clinical, social, and economic impacts of home parenteral nutrition dependence in short bowel syndrome. JPEN J Parenter Enteral Nutr. 2014;38(1 suppl):32S‐37S. 24418898 10.1177/0148607113517717

[13] Arhip L , Serrano‐Moreno C , Romero I , Camblor M , Cuerda C . The economic costs of home parenteral nutrition: systematic review of partial and full economic evaluations. Clin Nutr. 2021;40(2):339‐349. 32631611 10.1016/j.clnu.2020.06.010

[14] Winkler M , Tappenden K . Epidemiology, survival, costs, and quality of life in adults with short bowel syndrome. Nutr Clin Pract. 2023;38(suppl 1):S17‐S26. 37115027 10.1002/ncp.10964

[15] French C , Lal S , Jones D , et al. Impact of home parenteral nutrition on family members: a national multi‐centre cross‐sectional study. Clin Nutr. 2022;41(2):500‐507. 35007818 10.1016/j.clnu.2021.12.030

[16] Jeppesen PB , Chen K , Murphy R , Shahraz S , Goodwin B . Impact on caregivers of adult patients receiving parenteral support for short‐bowel syndrome with intestinal failure: a multinational, cross‐sectional survey. JPEN J Parenter Enteral Nutr. 2022;46(4):905‐914. 34368993 10.1002/jpen.2248 PMC9293039

[17] Smith CE , Piamjariyakul U , Yadrich DM , Ross VM , Gajewski B , Williams AR . Complex home care: part III‐‐economic impact on family caregiver quality of life and patients' clinical outcomes. Nurs econ. 2010;28(6):393‐414. 21291060 PMC3075108

[18] Berghöfer P , Fragkos KC , Baxter JP , et al. Development and validation of the disease‐specific Short Bowel Syndrome‐Quality of Life (SBS‐QoL™) scale. Clin Nutr. 2013;32(5):789‐796. 23274148 10.1016/j.clnu.2012.12.001

[19] Mirtallo JM , Blackmer A , Hennessy K , Allen P , Nawaya AD . Parenteral nutrition insecurity: ASPEN survey to assess the extent and severity of parenteral nutrition access and reimbursement issues. Nutr Clin Pract. 2024;39(2):396‐408. 38102986 10.1002/ncp.11110

[20] Mundi MS , Mercer DF , Iyer K , et al. Characteristics of chronic intestinal failure in the USA based on analysis of claims data. JPEN J Parenter Enteral Nutr. 2022;46(7):1614‐1622. 35726729 10.1002/jpen.2426

[21] Neumann ML , Allen JY , Ladner A , Kakani S , Weaver MS , Mercer DF . Exploring the impact of pediatric short bowel syndrome on parent well‐being using a disease‐specific pilot survey. Nutr Clin Pract. 2024;39(1):154‐167. 37245122 10.1002/ncp.11008

[22] Gutierrez SA , Pathak S , Raghu V , et al. Neighborhood income is associated with health care use in pediatric short bowel syndrome. J Pediatr. 2024;265:113819. 37940084 10.1016/j.jpeds.2023.113819 PMC10847979

[23] Chen Y , Yan M , Chen H , Sheng Y , Wang Z , Wu B . A systematic review of quality of life in patients with short bowel syndrome and their caregivers. Patient Prefer Adherence. 2024;18:1217‐1230. 38895637 10.2147/PPA.S443026 PMC11182759

[24] Van Oers HA , Haverman L , Olieman JF , et al. Health‐related quality of life, anxiety, depression and distress of mothers and fathers of children on home parenteral nutrition. Clin Nutr. 2019;38(4):1905‐1912. 30017244 10.1016/j.clnu.2018.06.981

[25] Beurskens‐Meijerink J , Huisman‐de Waal G , Wanten G . Evaluation of quality of life and caregiver burden in home parenteral nutrition patients: a cross‐sectional study. Clin Nutr ESPEN. 2020;37:50‐57. 32359755 10.1016/j.clnesp.2020.03.023

