# Supplementary material for: Short bowel syndrome–associated intestinal failure patient experience: A mixed‐method study leveraging an online patient community
Source: Nutr Clin Pract. 2025 Aug 7;40(5):1115–24. doi: 10.1002/ncp.70002 (PMC12450334; doi:10.1002/ncp.70002)
Supplement: Supplementary file 1 — inspire dg‐takeda sbs patient journey‐jan 20 2023 final clean NCP v2. [file NCP-40-1115-s001.docx]

# Discussion Themes

*Throughout the entirety of the session, the moderator will probe for emotions and sentiment around each topic. Participants will be asked to recall specific points in disease and treatment journey in preparation to answer recollection questions prior to interviews.*

**A. Introduction (10 minutes)**

The moderator will explain the purpose of the session and present the ground rules. The moderator will get an understanding of the patient as a high level.

**B. Diagnosis and Management/ Treatment (30 minutes)**

The moderator will ask questions regarding diagnosis of disease, management & treatment as well as HCPs involved in diagnosis and current care.

**C. Living with SBS (15 minutes)**

The moderator will ask questions regarding effects on Quality of Life (QoL).

**D. Opportunity Areas (5 minutes)**

The moderator will ask respondents what they believe is missing in currently offered treatment therapies, patient, or HCP education, or availability of patient support offerings that may improve their QoL, outlook on life, or minimize/reduce the burden of living with SBS.

# A. Introduction and Background [10 minutes]

This guide is not a script, and the moderator will maintain a conversational flow. This means that the moderator will jump around the guide as necessary and will mirror the lexicon that participants are using.

Thank you for joining us for this interview. I’m [moderator], and today we’re going to be discussing your experiences with short bowel syndrome, also known as SBS.

A few things to keep in mind as we start our conversation. We’re audio and video recording our session and several colleagues are listening in. Also, in the interest of protecting your privacy, please do not mention your last name, phone number, or address in our conversation today.

Today’s interview will take about 60 minutes, and because of time, I may occasionally interrupt you to keep us moving. You’re welcome to skip any questions you feel uncomfortable answering. Please keep in mind that there are no wrong answers to any of the questions I’m asking today. I’m here to learn about your experiences and opinions.

Some of the questions I ask today will be personal in nature, but rest assured that the intent of collecting this type of information is to better understand the challenges individuals living with conditions such as SBS can face and that we are not here to pass judgment. Your identity will remain confidential and be in no way associated with any findings associated with this research.

Lastly, if any side effects, negative reactions or complaints are mentioned about our client’s product(s), I’ll need to share that information with our client. Reporting this information will help our research sponsor learn more about the safety of their medicines and products. Our research sponsor may need to share that information with the United States government, their offices in other countries, and business partners. Any personal information about you or someone you mention will be handled confidentially. You also have the option to remain anonymous. Given all this, do you give me permission to give your name and contact information to our research sponsor’s Drug Safety Department ***if*** you mention a side effect, negative reaction, or complaint about one of their medicines or products during this interview?

- *[Gives permission]* Thank you.
- *[Does not give permission]* Okay, I will make sure your name and contact information are not shared.

**[Consent]** Given everything we’ve covered, are you okay to start the interview?

1. Tell me a little bit about yourself.
   1. [Moderator to share her screen] Which of the following best describes your housing situation?

| I do not have housing |
| --- |
| I have housing today, but I am worried about losing housing in the future |
| I have housing today and I am not worried about losing housing in the future |

- 1. [If patient does not have housing] Please explain to me your living situation.
  2. How many people live with you?
  3. Who are the people living in the household? PROBE alone, partner, spouse, family, friends, etc.
  4. Are you currently working? If so, what do you do for work?

1. Prior to your SBS diagnosis, did you have a doctor that you saw for general health check-ups? How close is the nearest hospital to you? Is this where you receive treatment (if currently on treatment)?
   - - - 1. In thinking about your SBS, where do you go to see the doctor for appointments? [PROBE hospital, office setting, etc.]

How do you get to your doctor appointments? How long does it take to get to these appointments?

Are you required to pay for parking when you see the doctor? If so, how much does that amount to each visit? [PROBE to understand financial burden on the patient]

- - - - 1. To what extent does transportation keep you from accessing medical needs? How about nonmedical needs?

1. What is your current understanding of your SBS? How would you describe it?

**B. Diagnosis and Treatment [30 minutes]**

*Next, I want to spend some time discussing your journey to get diagnosed with SBS.*

1. Approximately when were you initially diagnosed with SBS?
2. If you feel comfortable sharing, to what extent did a major life event cause your SBS diagnosis? [common causes of SBS in adults include cancer, damage to intestine caused by cancer treatment, Crohn’s disease, internal hernia, intestinal injury from loss of blood flow due to a blocked blood vessel, intestinal injury due to a motor vehicle accident, intestinal injury from trauma, midgut volvulus, intussusception (a.k.a., telescoping of intestine), complication of bariatric surgery (or another surgery), necrotizing enterocolitis (i.e., complication of prematurity), birth defect (e.g., gastroschisis, intestinal atresia, omphalocele, Hirschsprungs, etc…)]
   1. What was the specialty of the doctor who diagnosed your SBS?
   2. [Moderator to share her screen] Thinking about the past 12 months, how would you describe your general state of health on a scale of 1 – 5 where 1=very bad and 5=very good?
   3. Have you been diagnosed with any other gastrointestinal conditions related to your SBS, such as intestinal failure (i.e., intestinal failure where you’re requiring intravenous nutrition for fluid and/or nutrition)? PROBE some common conditions occurring among SBS patients include small bowel bacterial overgrowth (a.k.a. BO), liver problems (a.k.a. IFALD), kidney stones, central line infections, trouble maintaining weight.
3. Did your doctor refer you to another physician? If so, what type of doctor did the initial physician refer you to?
   - - - 1. About how much time had passed before you saw another doctor?
4. Thinking back at the beginning of your journey with SBS, ~~a~~bout how long did it take you to get the SBS diagnosis?
   1. What were your initial thoughts about your diagnosis?
   2. How did you feel after getting this diagnosis?
   3. How have these thoughts and feelings changed over time?
5. How did your physician explain that you have SBS? Please walk me through that conversation.
   1. How did the doctor tell you about the condition?
   2. What treatment or management options were discussed?
   3. How much time did your doctor spend discussing your treatment or management options?
   4. How did you feel about the information they gave you – did you feel overwhelmed or supported by it? Clear or difficult to understand?

*Now I’d like to talk to you about SBS treatment options and plans.*

1. Briefly walk me through the type(s) of treatment you have tried for your SBS. It’s okay not to mention specific product names. We are just trying to understand the steps you took that got you to where you are today. [LISTEN for mention of “intestinal rehab/rehabilitation”]
   1. How “easy” was it for you to start a treatment that worked well for you?
   2. What were your biggest concerns or fears when you first started this treatment?
      1. How, if at all, have your doctors/nurses/dietitians/care team helped to address those fears?
2. [If not mentioned as a treatment] Have you had surgery to increase the small intestine’s ability to absorb nutrients? Have you had intestinal transplant surgery?
   1. IF YES: How soon after your SBS diagnosis did you undergo surgery?
   2. What was the primary goal of surgery?
   3. What types of questions did you have about the surgery?
   4. What steps did you take after your surgery to continue to manage your SBS?
3. [If not mentioned as a treatment] To what extent have you and your doctor discussed parenteral nutrition (PN)? Enteral nutrition (EN)?
   - - - 1. What types of questions did you have regarding parenteral nutrition? Enteral nutrition?
         2. Who administers your parenteral nutrition? Enteral nutrition?

[If self-administering] Do you administer yourself or do you have someone assist you?

[If healthcare provider or caregiver administering] How often do you receive your parenteral nutrition? Enteral nutrition?

[If healthcare provider] How far is the office that administers your [parenteral / enteral] nutrition from your home?

How much time out of your day do the [parenteral / enteral] nutrition sessions typically take, including travel?

- - - - 1. How, if at all, has being on PN/EN changed your quality of life?

To what extent does being on PN/EN impact your ability to work, travel, attend social commitments, etc.?

1. [If patient is receiving PN] [Moderator to share her screen] Thinking about the parenteral nutrition that you’ve received over the past 12 months, how often did the injections follow the plan prescribed by your doctor? [Response options include Always, Sometimes, Never]
2. Outside of prescription medication, to what extent did your doctor suggest changes to your diet or were you referred to a nutritionist? What were their suggestions?
   1. [If mention of special diet] What types of foods and drinks did your doctor recommend for your diet? What does a typical day of breakfast, lunch and dinner look like?
      1. What types of foods and drinks did you remove from your diet? [PROBE if they removed alcohol from their diet]

[If still drinking] How many drinks would you say you consume per week?

To what extent does drinking alcohol impact your SBS? Impact your daily life?

- 1. How “easy” was it for you to implement changes to your diet?
     1. How, if at all, has this helped you to mitigate the symptoms of SBS?

1. Thinking about your current / most recent prescription treatment for SBS, how did your physician/care team explain the treatment options to you?
   - - - 1. Who discussed these options with you?
         2. To what extent did you think the advantages and disadvantages to treatment were clear?
         3. To what extent were you able to understand the treatment options as they were presented to you?
         4. What were you hoping to get from the treatment?
2. How effective is your current treatment at controlling your symptoms?
   1. To what extent are you experiencing pain due to your SBS?
      1. What do you use to manage the pain? Has your doctor ever prescribed you a pain medication?

IF YES: How, if at all, does pain medication help you to manage pain?

How often do you need to take pain medication to manage your pain?

Have you ever sought out other options for pain relief?

1. Not including side effects, how do you feel about your current treatment?
   1. PROBE: How it’s administered (e.g., intravenous, subcutaneous injection vs pill or pills at home)
      1. [If Intravenous or SubQ]. Do you administer your injections yourself or do you have someone assist you with injections?

[If self-administering] PROBE to understand burden on work, social life, travel, etc.

1. To what extent were you involved in the treatment decisions? Were you as involved as you wanted to be?
2. In what ways, if at all, did you include a spouse, family, friends, etc. in the treatment making process?
   - - - 1. What was their role?

To what extent did they attend visits? Find educational resources? Etc.

To what extent did you find them helpful?

1. With whom do you feel most comfortable discussing your treatment options and decisions with? (care team, partner, family, friends, other patients, etc.) Why?

**C. Living with SBS [15 minutes]**

*For the next portion of our conversation, I want to ask you about your experiences living with SBS. First, I’d like to get an understanding on some more personal experiences that may have happened within the past 12 months or are currently happening in your life. I’m going to share a few things on your screen and I’d like you to tell me which of them you may have experience with. Note that this is a standard list of items asked to all participants.*

| Broken major appliances (i.e., refrigerator, freezer, stove, air conditioning) |
| --- |
| No smoke detectors or smoke detectors that do not function properly |
| Uncontrolled water leaks |
| Mold or suspected mold |
| Lead paint or pipes in your home |
| Inadequate heat and/or hot water |
| Bugs or pests in your home that you are unable to exterminate |
| Stayed in an unsafe place and/or experienced homelessness |
| Had difficulty paying for food and/or were concerned that food would run out |
| Had difficulty paying for bills such as electricity, gas, oil, cell phone, water, etc. |
| Had difficulty paying for, or worried about paying for, copays, medication, procedures, etc. |
| None of the above |

1. [If mention difficulty paying for electricity, gas, etc.] In the past 12 months, has the electric, gas, oil, or water company threatened to cut off services to your home?
2. [If mention stayed in unsafe place] To what extent do you feel like your household, or your neighborhood environment is unsafe?
   1. Within the past 12 months, have you had any concerns for your personal safety?
3. [If mention difficulty paying for copays, medication, etc.] To what extent is paying medical bills a stressor for you?
4. [If mention difficulty paying for food] Within the past 12 months, have you worried that your food would run out before you had money to buy more?
   1. Within the past 12 months, had the food you bought not lasted long enough and didn’t have money to buy more?
5. Thinking about this list again, how have any of these acted as a barrier to accessing healthcare for your SBS?
6. To what extent are there aspects of your diagnosis or treatment that directly affect your family, friends, etc.? If yes, how are they affected?
   - - - 1. [If patient mentions spouse/partner] How, if at all, has your diagnosis impacted your relationship with your spouse/partner? [LISTEN for mentions of separation/divorce/counseling and probe accordingly]

How, if at all, has your diagnosis impacted your ability to be intimate with your partner/spouse?

- - - - 1. [If patient mentions children] How, if at all, has your diagnosis impacted your ability or desire to have children? Care for your children?

1. To what extent is your career trajectory impacted by your diagnosis or treatment? If yes, how is it affected? [LISTEN for inability to work]
   - - - 1. Thinking about your current job, how, if at all, does SBS impact your day-to-day? To what extent are you able to finish projects or tasks on time, attend regular meetings, etc.?
         2. How often, if at all, do you miss work to visit the doctor?

How accommodating is your job in allowing you to make your appointments?

- - - - 1. [If using employer’s insurance] To what extent does your current insurance plan cover your SBS treatment?

Has a need to find better insurance coverage for your treatment or care ever prompted you to search for new job or employment opportunity? Explain.

1. Now, I’d like to do a short sentence completion exercise with you. I will read the beginning of the sentence and then I would like you to complete the sentence with the first thing that comes to mind. I don’t want you to think too much about this, just your first thought or feeling.
   - - - 1. The most frustrating or challenging aspect of my SBS experience is _________________.
         2. If I could tell my family and friends one thing about my SBS journey, I would tell them _________________.
         3. If I could tell my doctor one thing about my SBS treatment experience, I would tell him/her ______________.
         4. For me, successful SBS treatment looks like ______________________.
2. Do you have someone who helps you with daily activities, relative to your SBS diagnosis?
   1. In what way does [person] provide support?
   2. How would you describe the impact that SBS has on [person]?
3. How, if at all, do the effects of SBS or the activities related to its management affect your ability to perform certain activities or participate in social events? [LISTEN for going out to eat and/or social events that include food/alcohol and probe accordingly]
   - - - 1. Describe for me a time your life has been impacted by your diagnosis. What was the event? How, if at all, did you have to adapt?
4. How, if at all, do the effects of SBS interfere with your outlook on the future?
5. Have you engaged with any support or advocacy groups relative to your SBS, its management, or the underlying condition that led to your SBS?
   - - - 1. [IF YES] What type of group or groups have you connected with?
         2. What were you seeking when you originally reached out or connected with the group?
         3. Did you get what you were looking for by connecting with the group? Did connecting with the group provide any benefit that you weren’t expecting?

**D. Conclusion [5 minutes]**

*We are almost out of time today, but we want to take these last few minutes to ask about any thoughts or experiences that you feel are important to this discussion that we have not covered.*

1. What, if anything, do you know now that you wish you knew when you received your SBS diagnosis?
2. Are there any therapies that you are aware of that you feel could improve your life, in any way?
   - - - 1. Why are you not using this therapy (access, cost, etc.)?
         2. How would this therapy improve your life, specifically?
         3. PROBE: Even if therapies are unknown to the respondent, probe on what part of their life they would want a therapy to improve.
3. What non-drug resources would you like to have available to you in order to help you manage your SBS?
   - - - 1. PROBE: Availability of adequate healthcare services (e.g., HCPs are not confident/capable of treating them, HCP education, lack of knowledge of latest treatments by regular HCPs, etc.)
         2. PROBE: Availability of social support/mental health services versed in patients with SBS
4. What else do you think we should be keeping in mind about the experience of living with SBS?

*Thank you for joining this session today. As a reminder, you’ll receive an honorarium as a token of our appreciation for joining us today. When I send you a thank you email, that’s a sign that Inspire is beginning to process that honorarium. We appreciate you participating in this interview with us!*
